# Associations of health and lifestyle factors with multidimensional quality of life among women with endometriosis: a cross-sectional study

**DOI:** 10.3389/fgwh.2026.1837763

**Published:** 2026-07-22

**Authors:** Marijana Matek Sarić, Marija Ljubičić, Tamara Sorić, Ana Sarić, Emili Marušić, Salma Abdi Mahmoud, Miran Čoklo

**Affiliations:** 1 Department of Health Studies, University of Zadar, Zadar, Croatia; 2 Nutrition Department, Psychiatric Hospital Ugljan, Ugljan, Croatia; 3 School of Medicine, Catholic University of Zagreb, Zagreb, Croatia; 4 Department of Pediatrics, General Hospital Zadar, Zadar, Croatia; 5 The State University of Zanzibar, School of Health and Medical Sciences, Zanzibar, Tanzania; 6 Institute for Anthropological Research, Zagreb, Croatia

**Keywords:** body mass index, dietary supplements, dyschezia, fertility impairment, patient-reported outcome measures, tobacco use

## Abstract

**Introduction:**

Endometriosis is a chronic disease associated with pain, fertility difficulties, and reduced quality of life (QoL). Beyond clinical symptoms, health-related and lifestyle behaviors may influence the long-term impact of the disease. This study aimed to assess multiple QoL dimensions in women with endometriosis and examine their associations with disease-related symptoms, body mass index (BMI), and lifestyle characteristics.

**Methods:**

A cross-sectional online survey was completed by 153 women aged ≥ 18 years with self-reported endometriosis. Data included sociodemographic characteristics, body weight, physical activity, diet, smoking, and digestive symptoms, including painful defecation. The Endometriosis Impact Questionnaire evaluated physical, psychological, social, sexual and intimate, fertility, educational, employment and financial, and lifestyle QoL across three recall periods. Non-parametric tests and multivariate linear regression models assessed group differences and factors associated with QoL dimensions and overall impact.

**Results:**

The greatest burden was observed in fertility, physical, and psychological dimensions, particularly during the previous 12 months. Body-weight change since symptom onset was associated with poorer outcomes across all QoL domains. BMI showed coding-dependent associations: continuous BMI predicted greater physical and psychological impact, whereas BMI ≥ 25.0 kg/m^2^ was linked to lower impact in physical (β=−0.42; *p* = 0.005), sexual and intimate (β=−0.31; *p* = 0.044), and employment and financial QoL (β=−0.31; *p* = 0.049). Painful defecation was associated with greater psychological and sexual and intimate impact and higher overall impact. Smoking was associated with greater lifestyle impact (β=0.38; *p* < 0.001) and higher overall impact (β=0.17; *p* = 0.042). Dietary supplement use was associated with lower fertility impact (β=−0.18; *p* = 0.049), whereas following a dietary regimen was associated with higher fertility impact (β=0.22; *p* = 0.014).

**Conclusion:**

Endometriosis substantially affects multiple life domains, particularly fertility and physical and psychological well-being. Weight change, painful defecation, and smoking were linked to poorer QoL, while BMI demonstrated complex domain-specific associations. Integrated medical and lifestyle approaches may improve long-term disease impact.

## 1 Introduction

Endometriosis is a chronic, estrogen-dependent, systemic, and inflammatory gynecological disease characterized by the presence of endometrial-like tissue outside the uterine cavity (1–5). It causes a wide range of symptoms, including chronic pelvic pain, dysmenorrhea, dyspareunia, dyschezia, and infertility (6, 7), which may be widespread and persist despite treatment (2, 8, 9). The condition affects approximately 10%–15% of women of reproductive age worldwide (1). Despite its prevalence, endometriosis is often described as a “missed disease” due to its unclear etiology and frequent diagnostic delays (4), partly because up to 20%–25% of women with endometriosis present with nonspecific symptoms or remain asymptomatic (10). Moreover, no single treatment currently provides a complete cure (3). Given its complex nature, endometriosis significantly affects physical, psychological, and social functioning, ultimately reducing quality of life (QoL).

The World Health Organization (WHO) defines QoL as an individual's perception of their position in life within the context of their culture, value systems, goals, expectations, and concerns (11). QoL encompasses multiple dimensions, including physical health, psychological state, functional independence, social relationships, belief systems, and environmental context (11). However, the impact of endometriosis extends beyond these categories (12–16). Research shows that the condition alters women's QoL across numerous dimensions, including physical, psychological, social, sexual and intimate functioning, fertility, work life, finances, education, and lifestyle (13, 17, 18). Recent evidence further emphasizes the importance of comprehensive, disease-specific QoL assessment tools capable of capturing these multidimensional effects and supporting patient-centered care (19).

Women with endometriosis frequently report a markedly reduced QoL compared with the general population (20). QoL outcomes may also vary according to socio-demographic, clinical, and psychological factors, highlighting the importance of examining multiple determinants of disease burden (21). Symptoms can limit daily functioning, sexual activity, intimate relationships, and work performance, contributing to frustration, anxiety, and depression (7). Chronic pain is a predominant symptom, occurring particularly during menstruation, during or after sexual intercourse, and during urination or defecation (3). Pain during defecation, with or without constipation, may result from bowel involvement, inflammation, or adhesions, and can substantially interfere with daily activities (22–25). Dyspareunia negatively affects sexual function and intimate relationships, increasing relationship-related stress and psychological burden (13). Fertility difficulties may also lead to emotional distress and reduced psychological well-being (26). Overall, chronic pain, infertility, and psychological factors such as anxiety and depression are consistently identified as key predictors of reduced QoL in women with endometriosis (27).

Although pain and reproductive difficulties are among the most extensively studied features of endometriosis, growing evidence indicates that factors such as body mass index (BMI), physical activity, dietary habits, and body image also influence health perceptions and overall QoL (28–33). A healthy lifestyle, incorporating regular physical activity, balanced nutrition, and effective stress management, is known to positively affect overall well-being (34, 35), yet the physical limitations and psychological burden associated with endometriosis may hinder the adoption or maintenance of such behaviors (36).

Despite increased research interest, the relationships between lifestyle variables, BMI, physical activity, unhealthy habits, and different dimensions of QoL remain insufficiently explored. Few studies have examined how disease manifestations and lifestyle factors interact to affect physical, psychological, social, reproductive, and sexual functioning, as well as work productivity, education, and financial status (8, 9, 28). Furthermore, the long-term impact of specific symptoms, such as pain during defecation, on daily functioning and QoL has not been thoroughly investigated (22–25). Alongside lifestyle behaviors, the use of dietary supplements has received attention due to their anti-inflammatory, antioxidant, antiproliferative, and immunomodulatory properties (37), although evidence regarding their effectiveness in improving QoL remains limited.

Although several validated instruments are available to assess health-related QoL in women with endometriosis, including the Endometriosis Health Profile-30 (EHP-30) (38) and its shorter version, the EHP-5 (39), the Endometriosis Impact Questionnaire (EIQ) was specifically developed to evaluate the long-term impact of endometriosis on women's lives (8). A distinctive feature of the EIQ is that it assesses disease impact across multiple recall periods, allowing a more comprehensive evaluation of the enduring burden of endometriosis over time. Given the multidimensional objectives of the present study, the EIQ was considered particularly suitable for capturing the wide-ranging effects of endometriosis on women's lives.For these reasons, it is essential to examine the associations between QoL in women with endometriosis and factors such as lifestyle behaviors, BMI, physical activity, dietary supplement use, and health-related symptoms including painful defecation. Understanding these relationships may clarify how such variables influence different QoL dimensions, including physical, psychological, social, and sexual functioning (8, 9, 28, 40).

Based on the existing literature, we hypothesized that lifestyle behaviors, BMI, and specific disease-related symptoms are associated with QoL in women with endometriosis. Higher BMI and weight gain since symptom onset, together with lower levels of physical activity, were expected to be related to poorer QoL across multiple dimensions. Conversely, regular physical activity and dietary supplement use were expected to correlate with better general health perceptions and emotional well-being. Furthermore, more severe and persistent symptoms, particularly pain during defecation, which may reflect deeper pelvic involvement, were assumed to contribute to greater functional impairment and psychosocial distress.

Thus, the primary aim of this study was to assess the physical, psychological, social, fertility-related, sexual and intimate, educational, employment and financial, as well as lifestyle dimensions of QoL among women with endometriosis. A further objective was to analyze the associations between these dimensions and perceived weight, disease-related symptoms, and lifestyle factors, and to identify the predictors most strongly associated with reduced overall QoL.

## 2 Materials and methods

### 2.1 Study participants

This cross-sectional study was conducted from 10th February 2025 to 1st July 2025. A total of 174 women with a self-reported history of endometriosis voluntarily participated. Participants were recruited using a non-probability convenience sampling approach through endometriosis patient associations, dedicated Facebook groups (both open and closed), and dissemination of the survey link via email, WhatsApp, and other electronic communication platforms. A snowball sampling method was also applied, whereby respondents were encouraged to share the survey with other women with endometriosis.

Inclusion criteria were age ≥ 18 years and self-reported history of endometriosis. This approach was adopted to maximize recruitment reach and inclusivity, as requiring diagnostic verification would have substantially restricted participation and potentially introduced selection bias. Exclusion criteria included male sex, absence of endometriosis, age < 18 years, and incomplete questionnaires. Three participants were excluded due to missing sociodemographic data, one due to missing height and weight information, and four because they did not indicate their country or region of residence. Participants living outside the Republic of Croatia (*N* = 13) were excluded from further analyses due to the small number and heterogeneity of this subgroup relative to the main sample. These excluded participants did not differ significantly from Croatian participants in sociodemographic characteristics, health-related variables, or main outcome measures. The final analytical sample consisted of 153 participants.

### 2.2 Ethical considerations

The study received ethical approval from the Ethics Committee of the University of Zadar, Croatia (approval number: 114-06/24-01/16; approval date: March 28, 2024) and was conducted in accordance with the principles of the Declaration of Helsinki and its revisions. Participation was voluntary, and informed consent was obtained from all participants before completing the questionnaire. The survey was anonymous, and no personally identifiable information was collected to ensure confidentiality.

### 2.3 Questionnaire

Sociodemographic data, medical history, lifestyle habits, and the perceived impact of endometriosis on QoL were collected through a self-administered online questionnaire. The questionnaire combined two original instruments: the Questionnaire on Health and Lifestyle Habits among Women with Endometriosis (41) and the EIQ (8). Written permission to use both questionnaires for research purposes was obtained from the respective authors.

An expanded version of the validated Questionnaire on Health and Lifestyle Habits among Women with Endometriosis (41) was developed. Compared to the original version, the questionnaire was extended and included items related to participants' education in fields potentially influencing baseline knowledge (e.g., health sciences, psychology, nutrition, physical activity), based on the assumption that formal training in these areas may shape relevant behaviors. Participants self-reported their body weight and height, frequency and duration of physical activity, number and timing of daily meals, and information on their use of dietary supplements and additives. Dietary habits were assessed through fruit, vegetable, and fluid intake, in accordance with the original instrument, and supplemented with questions on predominant dietary patterns (e.g., traditional, Mediterranean, vegan). Participants additionally reported their consumption of caffeine, alcohol, and tobacco products, as well as perceived endometriosis-related weight changes, adherence to specific dietary regimens, and awareness of the relationship between nutrition and symptom severity. Health-related questions also addressed digestive function, including stool frequency and consistency, pain during defecation, and perceptions of diet-related effects on digestive disturbances (41).

The EIQ was used to assess the long-term impact of endometriosis across eight QoL dimensions: physical, psychological, social, sexual and intimate, fertility, employment and financial, educational, and lifestyle, the latter assessing lifestyle-related coping behaviors, specifically the use of alcohol, tobacco, or other illicit substances to cope with endometriosis symptoms or their emotional impact (8). Each dimension was evaluated over three recall periods: the past 12 months, one to five years ago, and more than five years ago. Responses were rated on a 0–4 scale (0 = not at all, 1 = a little, 2 = somewhat, 3 = quite a lot, 4 = very much), with an additional option of 9 = not applicable, which was excluded from score calculations.

For each domain and recall period, scores were calculated as the sum of all applicable item responses divided by the maximum possible score (number of items × 4), then rescaled to a 0–100 scale, where 0 represents minimal and 100 maximal impact of endometriosis. Because higher EIQ scores indicate greater disease impact, positive regression coefficients reflect worse QoL (greater impact), whereas negative coefficients indicate lower perceived impact. The total score for each dimension was calculated as the mean across the three recall periods. If a participant did not provide responses for a specific dimension or recall period, a score for that part could not be generated. Both dimension-specific scores and the overall EIQ score were included in the final analysis (8).

### 2.4 Statistical analysis

The final sample comprised 153 women with endometriosis residing in Croatia. The Kolmogorov–Smirnov test was used to assess the normality of data distribution. Categorical variables were presented as absolute numbers and percentages, while numerical variables were summarized using the mean (M) or median (Mdn) and standard deviation (SD) or interquartile range (IQR), depending on their distribution.

Differences in recall scores across the three time periods within each QoL dimension were examined using the Friedman test. The Mann–Whitney U test was applied to numerical variables to explore between-group differences.

Multivariate linear regression analyses were performed to examine associations between variables. Several multivariable linear regression models were constructed for each QoL dimension (physical, psychological, social, sexual and intimate, fertility, employment and financial, educational, and lifestyle) and for the overall EIQ score. Predictor variables included age, education level, marital status, BMI, body weight changes, perceived body weight category, physical activity, following a dietary regimen, dietary supplement use, fluid intake, smoking, coffee and alcohol consumption, bowel problems, painful defecation, and constipation. BMI was analyzed both as a continuous variable (kg/m^2^) and as a dichotomous variable using a cut-off of 25.0 kg/m^2^ (<25.0 vs. ≥ 25.0 kg/m^2^), consistent with the WHO classification of overweight. The same set of predictor variables was entered simultaneously into each model, allowing assessment of the independent association of each predictor with the respective outcome while controlling for all other variables included in the model.

In observational studies examining QoL in women with endometriosis, multivariable models consistently show that clinical, psychosocial, and behavioral predictors explain a moderate proportion of variance in QoL outcomes. Studies identifying predictors of QoL in this population report meaningful standardized regression effects across symptom burden, emotional well-being, and health-related behaviors, corresponding to medium effect sizes within the Cohen framework (10, 42, 43). Therefore, a medium effect size (f^2^ = 0.15) was considered an appropriate and conservative estimate for the power calculation. An *a priori* power analysis was performed using the G*Power software (Heinrich Heine University Düsseldorf, Düsseldorf, Germany) to determine whether the planned sample size would provide adequate statistical power for a multiple linear regression model with 17 predictors. Assuming a medium effect size (f^2^ = 0.15), a significance level of *α* = 0.05, and a desired statistical power of 0.80, the analysis indicated that a minimum of 146 participants was required. After data collection, *a post hoc* power analysis was carried out for the final sample of 153 participants with 17 predictors, confirming that the achieved power was sufficient (1-*β* = 0.82).

Statistical analyses were conducted using SPSS Statistics version 21.0 (IBM, Armonk, NY, USA). A *p*-value < 0.05 was considered statistically significant.

## 3 Results

### 3.1 Sociodemographic, health-related, and lifestyle characteristics of women with endometriosis

The average age of participants was 34.8 years (SD = 5.9), with more than half (53.6%) between 26 and 35 years. Most participants were married or in a civil partnership (74.5%) and had a university-level education (75.8%) (Supplementary Table 1).

A change in body weight since the onset of endometriosis symptoms was reported by 66.0% of participants. Of these, 32.7% reported weight gain, 5.9% reported weight loss, and 27.4% reported fluctuating weight (Table 1). Nearly half of participants (48.4%) perceived themselves as overweight, and 22.9% reported following a dietary regimen. Bowel-related symptoms (including diarrhea, constipation, or mixed bowel habits) were present in 41.2% of participants, while pain during defecation was reported by more than half (58.2%) (Table 1).

**Table 1:** Health-related characteristics of the study group (*N* = 153).

Variable	Value
Body height (m), Mdn (IQR)	168.0 (8.0)
Body weight (kg), Mdn (IQR)	65.0 (14.0)
BMI, Mdn (IQR)	22.5 (5.7)
BMI category, N (%)	
< 18.5 kg/m^2^ (underweight)	8 (5.2)
18.5–24.9 kg/m^2^ (normal weight)	96 (62.7)
25.0–29.9 kg/m^2^ (overweight)	45 (29.4)
≥ 30.0 kg/m^2^ (obesity)	4 (2.6)
Perceived weight change since symptom onset, N (%)	
Weight gain	50 (32.7)
Weight loss	9 (5.9)
Weight fluctuation	42 (27.4)
No change	52 (34.0)
Perceived body weight, N (%)	
Underweight	12 (7.8)
Normal weight	67 (43.8)
Overweight	74 (48.4)
Following a dietary regimen, N (%)	
No	118 (77.1)
Yes	35 (22.9)
Bowel-related symptoms, N (%)	
Yes	63 (41.2)
No	90 (58.8)
Pain during defecation, N (%)	
Yes	89 (58.2)
No	64 (41.8)

Mdn, median; IQR, interquartile range; BMI, body mass index; N, number of participants.

The largest proportion of participants reported engaging in 1–3 h of physical activity per week (44.4%) (Table 2). Regarding dietary habits, 52.9% indicated partial adherence to a healthy diet, and the same proportion (52.9%) consumed fewer than three meals per day. The most commonly reported dietary patterns were the traditional diet (32.7%) and the Mediterranean diet (22.2%). A total of 77.1% of participants used dietary supplements, while 84.6% reported a daily fluid intake below 2000mL. Water was the predominant beverage choice (96.1%). Regarding substance use, 63.4% of participants were active smokers, 81.0% consumed coffee, and 50.3% reported occasional alcohol intake (Table 2).

**Table 2:** Lifestyle characteristics of the study group (*N* = 153).

Variable	Value
Physical activity, N (%)	
Less than 1 h per week	44 (28.8)
1–3 h per week	68 (44.4)
3–5 h per week	28 (18.3)
More than 5 h per week	13 (8.5)
Adherence to healthy eating habits, N (%)	
No	17 (11.1)
Yes	55 (35.9)
Partially	81 (52.9)
Number of meals per day, N (%)	
< 3 meals	81 (52.9)
≥ 3 meals	72 (47.1)
Predominant dietary pattern, N (%)	
Traditional diet	50 (32.7)
Mediterranean diet	34 (22.2)
Vegetarian and vegan	9 (5.9)
Gluten-free, lactose-free, and LCHF diet	10 (6.5)
Other/combinations	27 (17.6)
No particular attention to diet	23 (15.0)
Use of dietary supplements, N (%)	
Yes	118 (77.1)
No	35 (22.9)
Daily fluid intake, N (%)	
< 2,000 mL	129 (84.6)
> 2,000 mL	24 (15.4)
Most frequently consumed type of beverage, N (%)	
Water	147 (96.1)
Juices	6 (3.9)
Smoking, N (%)	
Yes	97 (63.4)
No	56 (36.6)
Coffee consumption, N (%)	
Yes	124 (81.0)
No	29 (19.0)
Alcohol consumption, N (%)	
Sometimes	77 (50.3)
Never	76 (49.7)

N, number of participants; LCHF, low-carbohydrate, high-fat.

### 3.2 Differences between quality-of-life dimensions related to endometriosis

Significant differences between recall periods were observed across all QoL dimensions except the educational dimension, with the strongest impact reported for the past 12 months (Table 3). When examining each period separately, as well as when combining scores across periods, the highest EIQ scores were observed in the fertility domain (Mdn = 66.6, IQR = 74.7), followed by the physical (Mdn = 61.6, IQR = 27.8), psychological (Mdn = 59.1, IQR = 42.0), and social dimensions (Mdn = 43.8, IQR = 46.4). The lifestyle dimension was the least affected (Mdn = 9.7, IQR = 33.3). The Mdn overall EIQ score was 36.2 (IQR = 27.8) (Table 3).

**Table 3:** Impact of endometriosis on quality-of-life dimensions across three recall periods (*N* = 153).

Quality-of-life dimension	Recall period			p^a^	Overall score
	Last 12 months	1 to 5 years ago	More than 5 years ago		
Biopsychosocial dimension, Mdn (IQR)					
Physical dimension	64.4 (46.3)	62.5 (34.6)	55.8 (32.7)	< 0.001	61.6 (27.8)
Psychological dimension	71.1 (54.3)	67.2 (40.2)	54.7 (54.3)	< 0.001	59.1 (42.0)
Social dimension	50.0 (60.9)	43.8 (54.7)	40.6 (62.5)	< 0.001	43.8 (46.4)
Sexual and intimate relationships, Mdn (IQR)	35.7 (39.3)	33.9 (46.4)	30.4 (67.9)	< 0.001	32.1 (34.5)
Fertility dimension, Mdn (IQR)	75.0 (87.5)	75.0 (70.8)	50.0 (100.0)	0.004	66.6 (74.7)
Employment and financial dimension, Mdn (IQR)	27.3 (31.8)	19.3 (34.1)	14.8 (42.6)	< 0.001	22.0 (33.0)
Educational dimension, Mdn (IQR)	0.0 (29.2)	0.0 (37.5)	14.6 (58.3)	0.439	9.7 (40.6)
Lifestyle dimension, Mdn (IQR)	0.0 (25.0)	4.2 (33.3)	0.0 (39.6)	0.010	9.7 (33.3)
Overall for all dimensions, Mdn (IQR)					36.2 (27.8)

a Friedman test.

Mdn, median; IQR, interquartile range; EIQ, Endometriosis Impact Questionnaire.

Among women who reported a change in body weight since the onset of endometriosis symptoms, a significant impact was observed across all QoL dimensions. The total mean rank was 87.2 (*p* < 0.001) for body weight change and 89.7 (*p* = 0.028) for perceived overweight (Table 4). Among smokers, endometriosis had a significant impact on the employment and financial dimension (mean rank = 76.7; *p* = 0.043) and on the lifestyle dimension (mean rank = 79.7; *p* < 0.001). In women who used dietary supplements, significant effects were found in the fertility (mean rank = 67.2; *p* = 0.038), employment and financial (mean rank = 75.9; *p* = 0.020), and lifestyle dimensions (mean rank = 70.5; *p* = 0.022). Painful defecation was associated with overall QoL dimensions, with a total mean rank of 83.4 (*p* = 0.034) (Table 4).

**Table 4:** Differences in the impact of endometriosis according to health-related and lifestyle characteristics (*N* = 153).

		QoL dimension								Overall QoL
Variable	Category / p-value	Physical	Psychological	Social	Sexual and Intimate	Fertility	Employment and Financial	Educational	Lifestyle	
Body weight change since symptom onset	yes	83.5	86.2	87.4	81.5	71.4	79.1	51.0	72.0	87.2
	no	64.5	59.1	56.8	55.8	48.3	56.6	34.3	56.5	57.1
	p^a^	0.012	< 0.001	< 0.001	0.001	0.001	0.002	0.003	0.021	< 0.001
Perceived overweight	yes	88.5	91.3	90.8	79.3	72.7	84.0	55.1	72.6	89.7
	no	72.6	71.5	71.8	70.7	60.1	66.6	41.3	64.3	72.2
	p^a^	0.048	0.014	0.018	0.276	0.084	0.023	0.023	0.261	0.028
Smoking	yes	81.7	81.0	82.3	71.9	62.6	76.7	46.9	79.7	82.7
	no	68.8	70.1	67.8	75.1	65.2	62.2	41.8	40.2	67.5
	p^a^	0.083	0.141	0.051	0.659	0.709	0.043	0.366	< 0.001	0.045
Physical activity less than 3 h per week	yes	81.8	80.3	81.2	72.3	62.4	72.7	45.2	66.8	79.3
	no	65.1	68.9	66.7	74.8	66.8	68.5	44.5	65.6	71.3
	p^a^	0.035	0.153	0.067	0.752	0.547	0.583	0.905	0.866	0.312
Following a dietary regimen	yes	80.4	81.5	83.0	74.3	81.8	78.3	50.5	80.5	89.5
	no	76.0	75.7	75.2	72.6	58.3	69.5	43.6	62.2	73.3
	p^a^	0.608	0.491	0.359	0.835	0.002	0.285	0.303	0.017	0.057
Dietary supplement use	yes	76.9	80.1	79.5	71.7	67.2	75.9	44.7	70.5	79.6
	no	77.5	66.5	68.5	77.5	51.3	56.9	46.0	52.8	68.1
	p^a^	0.939	0.110	0.198	0.487	0.038	0.020	0.854	0.022	0.175
Pain during defecation	yes	83.1	83.7	81.1	82.5	68.5	72.7	50.8	67.6	83.4
	no	68.6	67.6	71.4	60.3	56.4	69.8	36.8	65.0	68.0
	p^a^	0.046	0.027	0.182	0.002	0.065	0.683	0.010	0.699	0.034
Stool type	normal	71.8	72.1	71.8	68.8	60.9	67.5	44.6	65.7	73.9
	diarrhea	107.0	97.1	102.6	96.6	66.7	105.1	54.3	69.0	94.1
	constipation	84.3	92.5	88.7	65.7	65.7	73.3	30.5	78.2	81.0
	mixed	77.4	77.3	77.5	77.1	68.3	69.1	49.7	64.3	77.7
	p^b^	0.070	0.169	0.108	0.152	0.793	0.030	0.257	0.778	0.504

Values are presented as mean rank values calculated for the compared groups; .

a Mann–Whitney U test; .

b Kruskal Wallis test.

p, *p*-value.

### 3.3 Associations of health and lifestyle factors with the impact of endometriosis on quality-of-life dimensions

Strong positive correlations were observed among the QoL dimensions in women with endometriosis. The strongest association was between the psychological and social dimensions (Rs = 0.77; *p* < 0.001), with a similarly strong correlation between the social and employment and financial dimensions (Rs = 0.77; *p* < 0.001). A strong correlation was also observed between the physical and psychological dimensions (Rs = 0.72; *p* < 0.001) (Supplementary Table 2).

Linear regression analyses confirmed associations between body weight change since symptom onset and all QoL dimensions, with an overall impact score of *β* = 0.30 (*p* = 0.001), indicating greater perceived impact among women reporting weight change. BMI was positively associated with the physical (*β* = 0.53, *p* = 0.001) and psychological dimensions (*β* = 0.33, *p* = 0.033), suggesting that higher BMI values were related to greater disease impact in these dimensions. In contrast, BMI ≥ 25.0 kg/m^2^ was negatively associated with the physical (*β* = −0.42, *p* = 0.005), sexual and intimate (*β* = −0.31, *p* = 0.044), and employment and financial dimensions (*β* = −0.31, *p* = 0.049), indicating lower impact scores within this categorical comparison. Fertility was positively associated with following a dietary regimen (*β* = 0.22, *p* = 0.014) and negatively associated with dietary supplement use (*β* = −0.18, *p* = 0.049), suggesting higher perceived fertility-related impact among women following dietary regimens and lower impact among dietary supplement users. Smoking was positively associated with the lifestyle dimension (*β* = 0.38, *p* < 0.001) and with the overall impact of endometriosis on QoL (*β* = 0.17, *p* = 0.042), indicating greater lifestyle-related impact and poorer overall QoL among smokers. Painful defecation was linked to the psychological (*β* = 0.19, *p* = 0.023) and sexual and intimate dimensions (*β* = 0.30, *p* = 0.001), as well as to the overall impact score (*β* = 0.19, *p* = 0.024), reflecting higher perceived burden in these dimensions (Table 5).

**Table 5:** The associations between sociodemographic, health-related, and lifestyle characteristics and the impact of endometriosis on quality-of-life dimensions using multivariable linear regression models.

	QoL dimension																	
Variable	Physical		Psychological		Social		Sexual and Intimate		Fertility		Employment and Financial		Educational		Lifestyle		Overall QoL	
	*β*	p	β	p	β	p	β	p	β	p	β	p	β	p	β	p	β	p
Age	0.07	0.394	−0.08	0.297	0.05	0.533	0.03	0.672	0.00	0.999	0.10	0.229	0.06	0.606	0.08	0.323	−0.01	0.946
University degree	0.04	0.604	0.02	0.851	−0.04	0.631	−0.09	0.285	0.12	0.196	−0.10	0.255	−0.22	0.053	−0.06	0.459	0.01	0.890
Living with a partner	−0.05	0.555	0.04	0.630	0.00	0.966	0.06	0.496	0.02	0.853	0.04	0.682	0.07	0.552	0.02	0.804	0.06	0.460
BMI	0.53	0.001	0.33	0.033	0.15	0.339	0.26	0.093	0.04	0.773	0.27	0.098	−0.07	0.745	0.18	0.303	0.26	0.089
BMI ≥ 25.0 kg/m^2^	−0.42	0.005	−0.24	0.111	−0.14	0.353	−0.31	0.044	−0.03	0.858	−0.31	0.049	0.06	0.799	−0.23	0.168	−0.28	0.056
Body weight change since symptom onset	0.13	0.145	0.24	0.009	0.30	0.001	0.29	0.002	0.28	0.004	0.19	0.050	0.27	0.030	0.20	0.031	0.30	0.001
Perceived overweight	0.02	0.860	0.02	0.807	0.02	0.861	−0.01	0.928	−0.03	0.803	0.05	0.646	0.11	0.444	−0.03	0.729	0.01	0.945
Physical activity less than 3 h/week	0.12	0.135	0.07	0.401	0.10	0.203	−0.04	0.649	−0.08	0.374	−0.02	0.814	−0.04	0.705	−0.11	0.199	0.02	0.808
Following a dietary regimen	−0.03	0.748	−0.03	0.684	0.00	0.975	−0.01	0.950	0.22	0.014	0.03	0.764	0.05	0.670	0.13	0.137	0.06	0.435
Use of dietary supplements	0.05	0.509	−0.13	0.114	−0.09	0.275	0.04	0.638	−0.18	0.049	−0.12	0.179	−0.08	0.468	−0.14	0.096	−0.11	0.165
Daily fluid intake	−0.07	0.377	−0.01	0.889	−0.02	0.794	0.06	0.487	−0.01	0.933	−0.03	0.706	−0.06	0.578	−0.10	0.230	−0.03	0.707
Smoking	0.12	0.157	0.09	0.273	0.12	0.175	−0.01	0.935	−0.08	0.403	0.12	0.193	0.08	0.451	0.38	<0.001	0.17	0.042
No coffee	0.00	0.974	0.02	0.807	0.04	0.608	−0.02	0.847	−0.14	0.116	0.01	0.951	−0.05	0.665	−0.01	0.934	−0.06	0.459
No alcohol	0.08	0.354	0.04	0.637	0.00	0.955	0.13	0.119	−0.03	0.755	0.13	0.150	−0.17	0.147	−0.02	0.787	0.06	0.501
Bowel problems	0.11	0.222	0.05	0.598	0.06	0.540	−0.01	0.877	−0.06	0.532	0.12	0.205	−0.07	0.564	−0.03	0.782	−0.04	0.634
Painful defecation	0.15	0.088	0.19	0.023	0.10	0.262	0.30	0.001	0.17	0.072	0.01	0.876	0.20	0.104	0.08	0.347	0.19	0.024
Constipation	−0.07	0.399	−0.02	0.856	0.02	0.846	0.05	0.572	0.11	0.243	−0.04	0.661	0.02	0.893	−0.01	0.910	0.03	0.684

QoL, quality-of-life; β, beta coefficient; p, *p* value; BMI, body mass index.

Multivariable linear regression models were performed for each QoL dimension (one model per dimension), with all listed predictors included simultaneously in each model.

## 4 Discussion

This study provides a multidimensional assessment of QoL among women with endometriosis and identifies potentially modifiable health and lifestyle factors associated with disease burden. By examining physical, psychological, social, sexual and intimate, fertility, educational, employment and financial, and lifestyle dimensions across three recall periods, the findings extend existing literature beyond symptom severity and highlight the broader societal and functional impact of endometriosis. From a public health perspective, the results emphasize the need to integrate lifestyle-oriented and psychosocial components into routine endometriosis care.

Changes in body weight following symptom onset were associated with multiple QoL dimensions, indicating that body weight changes may represent an important indicator of disease burden. Most participants reporting body weight changes described weight gain or fluctuating weight, whereas relatively few reported weight loss. This pattern is noteworthy given that several previous studies have reported lower BMI among women with endometriosis compared with women without the disease (44, 45). However, BMI measured at a single time point and self-reported weight changes since symptom onset represent different constructs and should not be interpreted as directly comparable. Moreover, several factors, including chronic symptoms, treatment-related effects, and lifestyle modifications, may contribute to changes in body weight among women with endometriosis, although these factors were not assessed in the present study. Evidence suggests that body weight changes can affect physical functioning and psychological well-being (27), potentially exacerbating pain perception, fatigue, depressive symptoms, and social withdrawal (30, 31). Because the present study was cross-sectional and relied on retrospective self-report, the temporal relationship between endometriosis and body weight changes cannot be established, and these findings should therefore be interpreted with caution. These findings have implications for preventive and supportive care strategies, particularly in the context of the growing global prevalence of overweight and obesity. Structured weight management counseling, nutritional guidance, and psychological support could be incorporated into multidisciplinary care models to help women manage disease-related changes in body weight and their potential impact on QoL. At a systems level, this underscores the importance of embedding lifestyle counseling within gynecological and primary care services rather than treating it as an ancillary component.

BMI ≥ 25.0 kg/m^2^ was negatively associated with the physical, sexual and intimate, and employment and financial impact scores. Although the relationship between BMI and endometriosis remains inconsistent in the literature (33, 45), the present findings suggest that body weight may be related to differences in functional and social experiences, with associations varying depending on BMI coding. Higher BMI has been linked to reduced sexual activity and desire in longitudinal research (46, 47), while body image concerns may further affect intimate relationships (48). In our models, continuous BMI values were positively associated with greater physical and psychological impact, highlighting the complexity of these associations and the importance of nuanced interpretation. From a policy standpoint, addressing weight-related stigma, promoting healthy behaviors, and ensuring equitable access to lifestyle interventions may represent important components of comprehensive women's health strategies.

Painful defecation (dyschezia) was significantly associated with poorer psychological and sexual and intimate QoL. This finding reinforces the need to recognize gastrointestinal symptoms as central—not peripheral—to the lived experience of endometriosis. Prior research indicates that dyschezia and dyspareunia contribute to anxiety, stress, reduced perceived control, and impaired daily functioning (49–54). The present results further highlight the importance of actively engaging women in symptom management and care decisions, as patient engagement has been associated with improved coping, self-management, and QoL outcomes among individuals living with chronic pain conditions, including endometriosis (55). Sexual dysfunction is also frequently reported in women facing infertility, highlighting the interplay between reproductive concerns, physical discomfort, and intimate relationships (56). These symptoms may limit physical activity and occupational performance, thereby increasing indirect societal costs through reduced productivity. Improved screening for gastrointestinal symptoms within gynecological services and timely referral pathways may therefore have broader benefits for mental health, sexual well-being, and work participation.

The associations between dietary supplement use, dietary regimens, and the fertility dimension highlight the complex interplay between reproductive concerns and self-directed health behaviors. While supplement use was negatively associated with fertility impact scores, possibly reflecting lower perceived burden, following a dietary regimen was positively associated with fertility impact, suggesting that women experiencing greater reproductive difficulties may be more likely to seek dietary solutions. Nutritional interventions may influence oxidative stress, inflammation, and hormonal regulation (57), and dietary patterns may modulate estrogen levels and symptom expression (58). Additionally, engagement in dietary strategies may enhance perceived control and reduce stress related to fertility concerns (59). However, the cross-sectional design precludes causal conclusions. From a public health perspective, these findings support the development of evidence-based nutritional guidance tailored to women with endometriosis to prevent reliance on unverified or potentially ineffective interventions.

Psychological distress is prevalent among women with endometriosis and may impair emotional well-being, social functioning, and intimate relationships (60). The observed associations between body weight, symptoms, and multiple QoL dimensions further illustrate the interdependence of physical and mental health. Symptoms such as chronic pain, fatigue, and reduced concentration may adversely affect academic and occupational performance, increasing psychosocial stress and potentially contributing to financial strain (26). Consistent with the BMI-related findings discussed earlier, employment and financial experiences may be influenced by complex interactions between physical health, body perception, and social functioning. Collectively, these findings support the inclusion of mental health screening and counseling within standard endometriosis management protocols, as recommended in integrated care frameworks (16, 61, 62).

In the present study, 63.4% of participants reported active smoking, a prevalence substantially higher than that reported among women in the Croatian general population (21.7%) (63). Although the reasons for this finding cannot be determined from the present data, chronic pain, psychological distress, and disease-related stress are highly prevalent among women with endometriosis and may influence health-related behaviors, including smoking. However, because determinants of smoking behavior were not assessed in the present study, these explanations remain speculative and should be interpreted with caution.

Smoking emerged as a significant lifestyle factor associated with poorer lifestyle-related QoL and higher overall impact scores. Consistent with previous studies, smokers report worse outcomes across multiple QoL dimensions (64). Biologically, smoking may exacerbate endometriosis through increased oxidative stress, endocrine disruption, and chronic inflammation (65, 66). Smoking also negatively affects ovarian function and fertility by disrupting the hypothalamic–pituitary–ovarian axis and accelerating follicular atresia (67, 68), compounding reproductive challenges (69, 70). These findings reinforce smoking cessation as a priority in women's reproductive health strategies. Integrating targeted smoking cessation programs into gynecological and reproductive health services may yield benefits for both symptom management and long-term health outcomes.

This study has several limitations. The cross-sectional design limits causal inference, and reliance on self-reported endometriosis diagnosis and online data collection may introduce selection and recall bias. Information regarding the method of diagnosis (e.g., laparoscopic confirmation, imaging-based diagnosis, or clinical diagnosis) was not collected. Consequently, diagnostic heterogeneity cannot be excluded, which may affect the generalizability of the findings. Nevertheless, the use of self-reported diagnosis is common in large-scale survey-based studies of endometriosis, particularly when recruiting participants through patient associations and online communities. Additionally, the absence of detailed clinical indicators—such as disease stage, pain severity, comorbidities, medication use, and time since diagnosis—restricts clinical interpretation. Future longitudinal studies with clinically confirmed diagnoses and objective biomarkers are needed to clarify causal pathways and inform targeted interventions.

Despite these limitations, this study contributes to the growing recognition of endometriosis as a condition with substantial multidimensional and societal impact. By identifying modifiable lifestyle and health-related correlates of QoL, the findings support the development of integrated, multidisciplinary care models that combine medical treatment with lifestyle counseling, psychological support, and sexual health services. This approach is further supported by evidence indicating that more positive perceptions of quality of care are associated with better QoL outcomes among women with endometriosis, underscoring the importance of patient-centered and coordinated care pathways in endometriosis management (71). At the policy level, increased awareness, earlier diagnosis, and improved coordination between primary care, gynecology, mental health, and public health services may reduce long-term burden and improve quality of life for women with endometriosis (16, 61, 62).

The results underscore that endometriosis management should extend beyond symptom control to address behavioral, psychosocial, and reproductive dimensions. Strengthening preventive and supportive care strategies within women's health systems may help reduce disease-related inequities and enhance overall well-being.

## 5 Conclusion

This study demonstrates that endometriosis is associated with substantial multidimensional burden, particularly in the fertility, physical, and psychological dimensions of QoL. Painful defecation, body weight changes following symptom onset, and smoking were associated with poorer outcomes across several QoL dimensions, while BMI ≥ 25.0 kg/m^2^ was associated with lower impact scores in selected QoL dimensions, reflecting the complex and context-dependent nature of BMI-related findings. Dietary supplement use and following a dietary regimen were also associated with perceived fertility-related impact, underscoring the complex interplay between reproductive concerns and self-directed health strategies.

These findings reinforce the need to move beyond a symptom-focused model of care toward integrated, multidisciplinary approaches that address physical symptoms, mental health, sexual well-being, and lifestyle behaviors. Incorporating weight management support, smoking cessation interventions, nutritional counseling, and psychological assessment into routine gynecological care may contribute to improved long-term outcomes for women with endometriosis.

Given the cross-sectional design and reliance on self-reported data, causal inferences cannot be drawn. Future longitudinal research using clinically confirmed diagnoses and objective indicators of disease severity is needed to better understand causal pathways and to inform evidence-based clinical and public health strategies aimed at reducing the overall burden of endometriosis and improving women's QoL.

## Data Availability

The raw data supporting the conclusions of this article will be made available by the authors, without undue reservation.

## References

[1] Taylor HS Kotlyar AM Flores VA. Endometriosis is a chronic systemic disease: clinical challenges and novel innovations. Lancet. (2021) 397(10276):839–52. 10.1016/S0140-6736(21)00389-5 33640070

[2] Wei Y Zhao X Li L. The effect of circulating inflammatory proteins on endometriosis: a Mendelian randomization study. ImmunoTargets Ther. (2024) 13:585–93. 10.2147/ITT.S486139 39503011 PMC11537175

[3] World Health Organization. Endometriosis 2025. Available online at: https://www.who.int/news-room/fact-sheets/detail/endometriosis (Accessed December 11, 2025).

[4] The Lancet. Endometriosis: addressing the roots of slow progress. Lancet. (2024) 404(10460):1279. 10.1016/S0140-6736(24)02179-2 39368831

[5] Pehlivan MJ Sherman KA Wuthrich V Horn M Basson M Duckworth T. Body image and depression in endometriosis: examining self-esteem and rumination as mediators. Body Image. (2022) 43:463–73. 10.1016/j.bodyim.2022.10.012 36345084

[6] Coxon L Demetriou L Vincent K. Current developments in endometriosis-associated pain. Cell Rep Med. (2024) 5(10):101769. 10.1016/j.xcrm.2024.101769 39413731 PMC11513828

[7] Szypłowska M Tarkowski R Kułak K. The impact of endometriosis on depressive and anxiety symptoms and quality of life: a systematic review. Front Public Health. (2023) 11:1230303. 10.3389/fpubh.2023.1230303 37744486 PMC10512020

[8] Moradi M Parker M Sneddon A Lopez V Ellwood D. The endometriosis impact questionnaire (EIQ): a tool to measure the long-term impact of endometriosis on different aspects of women's Lives. BMC Womens Health. (2019) 19(1):64. 10.1186/S12905-019-0762-X 31088434 PMC6518659

[9] Cofini V Muselli M Petrucci E Lolli C Pelaccia E Guido M. Factors associated with chronic pelvic pain in women with endometriosis: a national study on clinical and sociodemographic characteristics, lifestyles, quality of life, and perceptions of quality of care, during the COVID-19 pandemic. Womens Health (Lond). (2024) 20:17455057241227361. 10.1177/17455057241227361 38449294 PMC10919124

[10] Pontoppidan K Olovsson M Grundström H. Clinical factors associated with quality of life among women with endometriosis: a cross-sectional study. BMC Womens Health. (2023) 23(1):551. 10.1186/S12905-023-02694-5 37875883 PMC10594903

[11] World Health Organization. Programme on Mental Health: WHOQOL User Manual, 2012 Revision (2012). Available online at: https://www.who.int/publications/i/item/WHO-HIS-HSI-Rev.2012-3 (Accessed December 12, 2025).

[12] Marinho MCP Magalhaes TF Fernandes LFC Augusto KL Brilhante AVM Bezerra LRPS. Quality of life in women with endometriosis. An Integrative Review. J Womens Health (Larchmt). (2018) 27(3):399–408. 10.1089/jwh.2017.6397 29064316

[13] Della Corte L Di Filippo C Gabrielli O Reppuccia S La Rosa VL Ragusa R. The burden of endometriosis on Women's Lifespan: a narrative overview on quality of life and psychosocial wellbeing. Int J Environ Res Public Health. (2020) 17(13):4683. 10.3390/ijerph17134683 32610665 PMC7370081

[14] Olliges E Bobinger A Weber A Hoffmann V Schmitz T Popovici RM. The physical, psychological, and social day-to-day experience of women living with endometriosis compared to healthy age-matched controls—a mixed-methods study. Front Glob Women's Health. (2021) 2:767114. 10.3389/fgwh.2021.767114/bibtex 34977863 PMC8714740

[15] Kufyah MT Salem TMA. Health-related quality of life in women with endometriosis; a systematic review. IJMDC. (2024) 8(1):379–86. 10.24911/ijmdc.51-1700386379

[16] Troìa L Luisi S. Sexual function and quality of life in women with endometriosis. Minerva Obstet Gynecol. (2022) 74(3):203–21. 10.23736/S2724-606x.22.05033-3 35420289

[17] Czubak P Herda K Niewiadomska I Putowski L Łańcut M Masłyk M. Understanding endometriosis: a broad review of its causes, management, and impact. Int J Mol Sci. (2025) 26(18):8878. 10.3390/ijms26188878 41009445 PMC12469443

[18] AlHarbi MH Almubarak RZ AlOmar SM Almazrou NH Fatani B Almubarak D. Quality of life of women with endometriosis: a systematic review. IJMDC. (2024) 8(12):3676–83. 10.24911/ijmdc.51-1704755106

[19] Manu A Poenaru E Duica F Bausic AIG Coroleuca BC Coroleuca CA. Quality of life assessment and clinical implications for women with endometriosis through validated tools: a narrative review. Medicina (Kaunas). (2025) 61(10):1729. 10.3390/medicina61101729 41155716 PMC12566188

[20] Gete DG Doust J Mortlock S Montgomery G Mishra GD. Impact of endometriosis on women's health-related quality of life: a national prospective cohort study. Maturitas. (2023) 174:1–7. 10.1016/J.maturitas.2023.04.272 37182389

[21] Bień A Pokropska A Grzesik-Gąsior J Korżyńska-Piętas M Pieczykolan A Zarajczyk M. Quality of life in women with endometriosis: the importance of socio-demographic, diagnostic-therapeutic, and psychological factors. J Clin Med. (2025) 14(12):4268. 10.3390/jcm14124268 40566013 PMC12194382

[22] Wolthuis AM Meuleman C Tomassetti C D'Hooghe T de Buck van Overstraeten A D'Hoore A. Bowel endometriosis: colorectal surgeon's Perspective in a multidisciplinary surgical team. World J Gastroenterol. (2014) 20(42):15616–23. 10.3748/wjg.V20.I42.15616 25400445 PMC4229526

[23] Dunleavy KA Ramos GP Dilmaghani S Green I Fadadu P Cope A. S535 evaluating irritable bowel syndrome symptoms and pelvic floor dysfunction in patients with deep endometriosis of the posterior pelvis. Am J Gastroenterol. (2022) 117(10S):e377–8. 10.14309/01.ajg.0000858780.40602.9b

[24] Kanthimathinathan V Elakkary E Bleibel W Kuwajerwala N Conjeevaram S Tootla F. Endometrioma of the large bowel. Dig Dis Sci. (2007) 52(3):767–9. 10.1007/S10620-006-9623-1 17268828

[25] Ferrero S Moioli M Dodero D Barra F. Symptoms of bowel endometriosis. In: Ferrero S Ceccaroni M, editors. Clinical Management of Bowel Endometriosis. Cham: Springer Nature Switzerland AG (2020):33–9. 10.1007/978-3-030-50446-5_4

[26] Maulenkul T Kuandyk A Makhadiyeva D Dautova A Terzic M Oshibayeva A. Understanding the impact of endometriosis on women's Life: an integrative review of systematic reviews. BMC Womens Health. (2024) 24(1):524. 10.1186/S12905-024-03369-5 39300399 PMC11411992

[27] Netzl J Gusy B Voigt B Sehouli J Mechsner S. Physical and psychosocial factors are crucial for maintaining physical and mental health in endometriosis: a longitudinal analysis. Psychol Health. (2025) 40(7):1156–77. 10.1080/08870446.2024.2302486 38251641

[28] Habib N Buzzaccarini G Centini G Moawad G Ceccaldi PF Gitas G. Impact of lifestyle and diet on endometriosis: a fresh look to a busy corner. Prz Menopauzalny. (2022) 21(2):124–32. 10.5114/pm.2022.116437 36199735 PMC9528818

[29] Matek Sarić M Sorić T Sarić A Marušić E Čoklo M Mavar M. The role of plant-based diets and personalized nutrition in endometriosis management: a review. Medicina (B Aires). (2025) 61(7):1264. 10.3390/medicina61071264 PMC12299565 40731893

[30] Hong J Yi KW. What is the link between endometriosis and adiposity? Obstet Gynecol Sci. (2022) 65(3):227–33. 10.5468/ogs.21343 35081675 PMC9119730

[31] Tang Y Zhao M Lin L Gao Y Chen GQ Chen S. Is body mass index associated with the incidence of endometriosis and the severity of dysmenorrhoea: a case–control study in China? BMJ Open. (2020) 10(9):e037095. 10.1136/bmjopen-2020-037095 32895278 PMC7478014

[32] Mollazadeh S Najmabadi KM Mirghafourvand M Khadivzadeh T Moghri J Hafizi L. The health-promoting lifestyle and its relationship with the impacts of endometriosis on women's Lives in Iran, 2022: a cross-sectional study. BMC Womens Health. (2025) 25(1):153. 10.1186/S12905-025-03696-1 40176003 PMC11963405

[33] Rowlands IJ Hockey R Abbott JA Montgomery GW Mishra GD. Body mass index and the diagnosis of endometriosis: findings from a national data linkage cohort study. Obes Res Clin Pract. (2022) 16(3):235–41. 10.1016/j.orcp.2022.04.002 35431154

[34] Vilchez-Chavez AF Bernal Altamirano E Morales-García WC Sairitupa-Sanchez L Morales-García SB Saintila J. Healthy habits factors and stress associated with health-related quality of life in a Peruvian adult population: a cross-sectional study. J Multidiscip Healthc. (2023) 16:2691–700. 10.2147/jmdh.S412962 37720270 PMC10505026

[35] Martland RN Ma R Paleri V Valmaggia L Riches S Firth J. The efficacy of physical activity to improve the mental wellbeing of healthcare workers: a systematic review. Ment Health Phys Act. (2024) 26:100577. 10.1016/j.mhpa.2024.100577

[36] Kovács-Szabó Z Ács P Prémusz V Makai A Hock M. Psychological, symptom-related, and lifestyle predictors of health-related quality of life in Hungarian women with endometriosis. J Clin Med. (2025) 14(19):7004. 10.3390/jcm14197004 41096084 PMC12524366

[37] Julio T Fenerich BA Halpern G Carrera-Bastos P Schor E Kopelman A. The effects of oral nutritional supplements on endometriosis-related pain: a narrative review of clinical studies. J Gynecol Obstet Hum Reprod. (2024) 53(10):102830. 10.1016/j.jogoh.2024.102830 39067786

[38] Jones G Kennedy S Barnard A Wong J Jenkinson C. Development of an endometriosis quality-of-life instrument: the endometriosis health profile-30. Obstet Gynecol. (2001) 98(2):258–64. 10.1016/s0029-7844(01)01433-8 11506842

[39] Jones G Jenkinson C Kennedy S. Development of the short form endometriosis health profile questionnaire: the EHP-5. Qual Life Res. (2004) 13(3):695–704. 10.1023/B:QURE.0000021321.48041.0e 15130031

[40] Bahat PY Ayhan I Özdemir EÜ İnceboz Ü Oral E. Dietary supplements for treatment of endometriosis: a review. Acta Biomed. (2022) 93(1):e2022159. 10.23750/abm.V93I1.11237 35315418 PMC8972862

[41] Tomić B. Dietary Habits and Bowel Elimination in Patients with Endometriosis According to M. Gordon's Functional Health Pattern [thesis]. Rijeka: University of Rijeka, Faculty of Health Studies (2021). Available online at: https://repository.fzsri.uniri.hr/islandora/object/fzsri:1291 (Accessed December 11, 2025).

[42] Wu YH Lu YY Liu KF. Factors influencing health-related quality of life in women with endometriosis: a cross-sectional study. Nurs Health Sci. (2024) 26(1):e13100. 10.1111/nhs.13100 38374495

[43] Kupec T Kennes LN Senger R Meyer-Wilmes P Najjari L Stickeler E. The multifactorial burden of endometriosis: predictors of quality of life. J Clin Med. 2025 14(2):323. 10.3390/jcm14020323 39860327 PMC11765614

[44] Desai SN Reed CC Mendez Y Yang Q Guan X. The hidden link between endometriosis and obesity: a state-of-the-art review. Cureus. (2026) 18(2):e102896. 10.7759/cureus.102896 41798441 PMC12962185

[45] Holdsworth-Carson SJ Dior UP Colgrave EM Healey M Montgomery GW Rogers PAW. The association of body mass index with endometriosis and disease severity in women with pain. J Endometr Pelvic Pain Disord. (2018) 10(2):79–87. 10.1177/2284026518773939

[46] van Poll M van Barneveld E Aerts L Maas JWM Lim AC de Greef BTA. Endometriosis and sexual quality of life. Sex Med. (2020) 8(3):532–44. 10.1016/j.esxm.2020.06.004 32712127 PMC7471125

[47] Nackers LM Appelhans BM Segawa E Janssen I Dugan SA Kravitz HM. Associations between body mass index and sexual functioning in midlife women: the study of Women's Health across the nation (SWAN). Menopause. (2015) 22(11):1175–81. 10.1097/gme.0000000000000452 25803669 PMC4580485

[48] Sullivan-Myers C Sherman KA Beath AP Cooper MJW Duckworth TJ. Body image, self-compassion, and sexual distress in individuals living with endometriosis. J Psychosom Res. (2023) 167:111197. 10.1016/j.jpsychores.2023.111197 36805454

[49] Norinho P Martins MM Ferreira H. A systematic review on the effects of endometriosis on sexuality and couple's Relationship. Facts Views Vis Obgyn. (2020) 12(3):197–205. 33123695 PMC7580264

[50] Aerts L Grangier L Streuli I Dällenbach P Marci R Wenger J. Psychosocial impact of endometriosis: from co-morbidity to intervention. Best Pract Res Clin Obstet Gynaecol. (2018) 50:2–10. 10.1016/j.bpobgyn.2018.01.008 29545113

[51] Da Anunciação IVN Benício MJdS Oliveira De Melo AB Mascari AC Laboissiere MEF Sousa LKL. Impacto da endometriose na qualidade de vida e na saúde mental de mulheres em idade reprodutiva: uma revisão integrativa [impact of endometriosis on the quality of life and mental health of women of reproductive age: an integrative review]. Braz J Implantol Health Sci. (2025) 7(8):407–16. 10.36557/2674-8169.2025v7n8p407-416

[52] Baetas BV Bretas BV Maziviero CM De Moraes GZ Rodrigues LTS Zanluchi A. Endometriose e a qualidade de vida das mulheres acometidas [endometriosis and quality of life of the women affected]. Rev Eletrônica Acervo Cient. (2021) 19:e5928. 10.25248/reac.e5928.2021

[53] Fiala L Lenz J Adamik Z Sajdlova R Kestlerova D Vetvicka V. Psychological hallmarks of endometriosis with emphasis on sexual dysfunction, stress, anxiety and depressive symptoms. Int Clin Pathol J. (2023) 10(1):45–8. 10.15406/icpjl.2023.10.00218

[54] Warzecha D Szymusik I Wielgos M Pietrzak B. The impact of endometriosis on the quality of life and the incidence of depression-A cohort study. Int J Environ Res Public Health. (2020) 17(10):3641. 10.3390/ijerph17103641 32455821 PMC7277332

[55] Ahmad M Sechi C Guerriero S Guicciardi M Vismara L. Patient engagement, quality of life and chronic pain: a cross-sectional study on endometriosis. J Health Psychol. (2025) 30(8):1990–2000. 10.1177/13591053241302134 39628115

[56] Amza M Sima RM Conea IM Bobei TI Augustin FE Pleş L. The impact of endometriosis on patients' Quality of sexual life. J Med Life. (2025) 18(2):90–3. 10.25122/jml-2024-0262 40134441 PMC11932512

[57] Barrea L Verde L Annunziata G Chedraui P Petraglia F Cucalón G. Effectiveness of medical nutrition therapy in the management of patients with obesity and endometriosis: from the Mediterranean diet to the ketogenic diet, through supplementation. The role of the nutritionist in clinical management. Curr Obes Rep. (2025) 14:68. 10.1007/S13679-025-00662-8 40920291 PMC12417264

[58] Zhou L Liu B Jian X Jiang L Liu K. Effect of dietary patterns and nutritional supplementation in the management of endometriosis: a review. Front Nutr. (2025) 12:1539665. 10.3389/fnut.2025.1539665 40144566 PMC11937854

[59] Alrashidi AS Feraih Aljaghwani L Saleh AlMohimeed R. The effect of nutrient supplementation on female fertility: a systematic review. Cureus. (2024) 16(8):e67028. 10.7759/cureus.67028 39280553 PMC11402477

[60] Kalfas M Chisari C Windgassen S. Psychosocial factors associated with pain and health-related quality of life in endometriosis: a systematic review. Eur J Pain. (2022) 26(9):1827–48. 10.1002/ejp.2006 35802060 PMC9543695

[61] Nowocin P Kudas Z Koszyk M Litwin A Krzywicka K Wiktor Kulczyński D. The impact of endometriosis on women's Mental health and quality of life – review. Med Sci. (2024) 28(154):e160ms3483. 10.54905/disssi.V28I154.E160MS3483

[62] Ribeiro SL Carranza JSS da Silva Pereira JI de Fátima V da Motta JPC Crepory MM. Abordagens multidisciplinares no manejo da endometriose [multidisciplinary approaches in the management of endometriosis]. Contrib a las Ciencias Soc. (2024) 17(8):1–14. 10.55905/revconv.17N.8-102

[63] Padjen I Dabić M Glivetić T Biloglav Z Biočina-Lukenda D Lukenda J. The analysis of tobacco consumption in Croatia–are we successfully facing the epidemic? Cent Eur J Public Health. (2012) 20(1):5–10. 10.21101/cejph.a3702 22571009

[64] Muselli M Mancinelli M Limoncin E Lolli C Pelaccia E Guido M. Investigating unhealthy behaviors associated with SF-36 domains in women with endometriosis—findings from a web-based survey data set. Behav Sci. (2024) 14(3):199. 10.3390/bs14030199/S1 38540502 PMC10968121

[65] Clower L Fleshman T Geldenhuys WJ Santanam N. Targeting oxidative stress involved in endometriosis and its pain. Biomolecules. (2022) 12(8):1055. 10.3390/biom12081055 36008949 PMC9405905

[66] Vallée A Feki A Josseran L Ayoubi JM. Smoking and endometriosis: a narrative review. Tob Induc Dis. (2025) 23:1–24. 10.18332/tid/203429 PMC12199790 40575735

[67] Oladipupo I Ali T Hein DW Pagidas K Bohler H Doll MA. Association between cigarette smoking and ovarian reserve among women seeking fertility care. PLoS One. (2022) 17(12):e0278998. 10.1371/journal.pone.0278998 36512605 PMC9746951

[68] Kline J Tang A Levin B. Smoking, alcohol and caffeine in relation to two hormonal indicators of ovarian age during the reproductive years. Maturitas. (2016) 92:115–22. 10.1016/j.maturitas.2016.07.010 27621248

[69] Lu R Jiao W Li X Zeng C Shang J Xue Q. Ovarian endometriosis negatively impacts pregnancy outcomes in young infertile women undergoing IVF/ICSI treatment. Eur J Med Res. (2025) 30(1):555. 10.1186/S40001-025-02816-9 40604864 PMC12218940

[70] Cui J Wang Y. Premature ovarian insufficiency: a review on the role of tobacco smoke, its clinical harm, and treatment. J Ovarian Res. (2024) 17(1):8. 10.1186/S13048-023-01330-Y 38191456 PMC10775475

[71] Cofini V Muselli M Lolli C Fabiani L Necozione S. Does quality of care (QoC) perception influence the quality of life (QoL) in women with endometriosis? Results from an Italian nationwide survey during COVID pandemic. Int J Environ Res Public Health. (2022) 20(1):625. 10.3390/ijerph20010625 36612945 PMC9819574

