# Supplementary material for: Associations of health and lifestyle factors with multidimensional quality of life among women with endometriosis: a cross-sectional study
Source: Front Glob Womens Health. 2026 Jul 22;7:1837763. doi: 10.3389/fgwh.2026.1837763 (PMC13437628; doi:10.3389/fgwh.2026.1837763)
Supplement: Supplementary file 1 [file Table1.docx]

Supplementary Material

**Supplementary Table 1.** Sociodemographic characteristics of the study group (N = 153).

| **Variable** | **Value** |
| --- | --- |
| Age (M, SD) | 34.8 (5.9) |
| Age group (N, %) |  |
| ≤ 25 years | 11 (7.2) |
| 26-35 years | 82 (53.6) |
| 36-45 years | 55 (35.9) |
| 46-48 years | 5 (3.3) |
| Marital status (N, %) |  |
| Married/Civil partnership | 114 (74.5) |
| Not married/divorced | 39 (25.5) |
| Education level (N, %) |  |
| High school | 37 (24.2) |
| University | 116 (75.8) |

**Abbreviations:** M, mean value; SD, standard deviation; N, number of participants.

**Supplementary Table 2.** Correlations between quality-of-life dimensions in women with endometriosis.

| **QoL dimension** | | **QoL dimension** | | | | | | |
| --- | --- | --- | --- | --- | --- | --- | --- | --- |
|  |  | **Psychological** | **Social** | **Sexual and Intimate** | **Fertility** | **Employment and Financial** | **Educational** | **Lifestyle** |
| Physical | Rs | 0.72 | 0.68 | 0.50 | 0.30 | 0.66 | 0.48 | 0.36 |
|  | p | < 0.001 | < 0.001 | < 0.001 | 0.001 | < 0.001 | < 0.001 | < 0.001 |
| Psychological | Rs |  | 0.77 | 0.54 | 0.49 | 0.69 | 0.44 | 0.39 |
|  | p |  | < 0.001 | < 0.001 | <0.001 | < 0.001 | < 0.001 | < 0.001 |
| Social | Rs |  |  | 0.58 | 0.28 | 0.77 | 0.56 | 0.40 |
|  | p |  |  | < 0.001 | 0.001 | < 0.001 | < 0.001 | < 0.001 |
| Sexual and Intimate | Rs |  |  |  | 0.38 | 0.59 | 0.59 | 0.25 |
|  | p |  |  |  | <0.001 | < 0.001 | < 0.001 | 0.006 |
| Fertility | Rs |  |  |  |  | 0.30 | 0.41 | 0.25 |
|  | p |  |  |  |  | 0.001 | < 0.001 | 0.009 |
| Employment and Financial | Rs |  |  |  |  |  | 0.59 | 0.53 |
|  | p |  |  |  |  |  | < 0.001 | < 0.001 |
| Educational | Rs |  |  |  |  |  |  | 0.46 |
|  | p |  |  |  |  |  |  | < 0.001 |

**Abbreviations:** QoL, quality-of-life; Rs, Spearman's rho coefficient correlation; p, p-value.
